# Factors affecting distribution patterns of organic carbon in sediments at regional and national scales in China

**DOI:** 10.1038/s41598-017-06035-z

**Published:** 2017-07-14

**Authors:** Qingqing Cao, Hui Wang, Yiran Zhang, Rattan Lal, Renqing Wang, Xiuli Ge, Jian Liu

**Affiliations:** 1 Institute of Environmental Research, Shandong University, Jinan, 250100 China; 2 Carbon Management and Sequestration Center, The Ohio State University, Columbus, 43210 USA; 3 School of Life Sciences, Shandong University, Jinan, 250100 China; 4 Shenyang Academy of Environmental Sciences, Shenyang, 110167 China; 5 Shandong Provincial Engineering and Technology Research Center for Vegetation Ecology, Shandong University, Jinan, 250100 China; 6 School of Environmental Science and Engineering, Qilu University of Technology, Jinan, 250353 China

## Abstract

Wetlands are an important carbon reservoir pool in terrestrial ecosystems. Light fraction organic carbon (LFOC), heavy fraction organic carbon (HFOC), and dissolved organic carbon (DOC) were fractionated in sediment samples from the four wetlands (ZR: Zhaoniu River; ZRCW: Zhaoniu River Constructed Wetland; XR: Xinxue River; XRCW: Xinxue River Constructed Wetland). Organic carbon (OC) from rivers and coasts of China were retrieved and statistically analyzed. At regional scale, HFOC stably dominates the deposition of OC (95.4%), whereas DOC and LFOC in ZR is significantly higher than in ZRCW. Concentration of DOC is significantly higher in XRCW (30.37 mg/l) than that in XR (13.59 mg/l). DOC and HFOC notably distinguish between two sampling campaigns, and the deposition of carbon fractions are limited by low nitrogen input. At the national scale, OC attains the maximum of 2.29% at precipitation of 800 mm. OC has no significant difference among the three climate zones but significantly higher in river sediments than in coasts. Coastal OC increases from Bohai Sea (0.52%) to South Sea (0.70%) with a decrease in latitude. This study summarizes the factors affecting organic carbon storage in regional and national scale, and have constructive implications for carbon assessment, modelling, and management.

## Introduction

Increasing in atmospheric concentration of carbon dioxide (CO_2_) and methane (CH_4_) since mid-20^th^ century^1^ causes the global warming^2^. Wetland ecosystems can deposit a large amount of photosynthesized carbon (C) into sediments^3^, and in-depth research on organic carbon (OC) sequestration and distribution must be undertaken to understand the processes and factors affecting it. OC can be divided into three C fractions on the basis of the stability and solubility of C in soils or sediments^4^. These fractions are: heavy fraction organic carbon (HFOC), light fraction organic carbon (LFOC) and dissolved organic carbon (DOC). Among the three fractions, HFOC (density >  = 1.7 g cm^−3^) is relatively stable to climate change and other external environmental conditions^5^, and LFOC (density <  = 1.7 g cm^−3^) is sensitive to the change of environment and microbial activities^6^. Furthermore, DOC has been studied widely with regards to biochemical activities, such as nitrification and denitrification^7^, and C mineralization^8^. Thus, carbon storage and factors affecting the carbon storage can be indicated by the study on carbon fractions as they participate in many biochemical activities and are easily affected by environmental variables.

Wetland, with its abundant plants and microbes, has a higher capacity of C deposition than cultivated soils or other land types^9^. River, as one natural wetland, can deposit plant decays and denature pollutants. To improve the efficiency and accelerate these processes, wetlands have been constructed near the river wetlands^10^. Thus, it is important to study whether constructed wetland has larger preponderance in C deposition than in the river wetland.

At regional scale, Cao *et al*. (2015) indicated that constructed wetland has higher carbon storage than river wetland^4^. Previous reports showed that surface soil has higher concentrations than subsurface soil^11, 12^. Guo *et al*. (2015) shows the microbial phylum *Acidobacteria* can inhibit the decomposition and mineralization of organic carbon^13^. And Xu (2015) also showed the advantages of summer on carbon mineralization over winter in wetland^14^. At large scale, Mitsch *et al*. (2014) reported that tropical wetlands have significantly higher OC than boreal wetlands^15^, and variation in precipitation, climate and landscape can also influence the C distribution and storage^16, 17^. Therefore, the regional and large-scale factors such like wetland types, soil depths, seasons, climate and precipitation, etc. may affect the OC deposition more or less. It is pertinent to identify important factors in the assessment of C storage at regional and national scales. However, systematic analysis of these factors affecting wetland C storage has not been undertaken in China.

Therefore, a research project was implemented at regional and national scale to study the factors affecting deposition of OC in wetland. The principal objectives of this study were to: 1) Assessing the distribution difference of three C fractions in two wetland types, two sampling campaigns, and among the sampling stations, 2) Evaluating the distribution of OC at the national scale, and, 3) Determining the relevant factors (precipitation, nitrogen content, microbes, etc.) affecting the distribution and storage of OC in wetland ecosystems. These objectives are realized by testing the hypothesis that sampling season and wetland types can significantly affect the storage of C fractions at regional scale, and OC is also affected by precipitations and climatic zones at large scale.

## Results

### Distribution patterns of carbon fractions at regional scale

**Figure 1:**
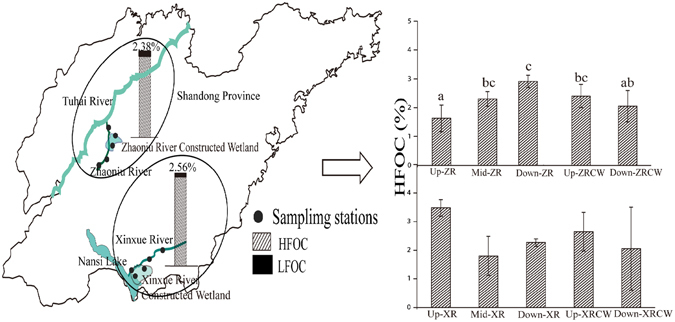
Sampling areas and C contents in Shandong Province (HFOC: heavy fraction organic carbon. LFOC: light fraction organic carbon). Up/Mid/Down-ZR: upstream/midstream/downstream of Zhaoniu River. Up/Down-ZRCW: upstream/downstream of Zhaoniu River Constructed Wetland. Up/Mid/Down-XR: upstream/midstream/downstream of Xinxue River. Up/Down-XRCW: upstream/downstream of Xinxue River Constructed Wetland. Bars sharing the same lowercase letter (a or b) are not significant at α = 0.05 (Duncan test). XR and XRCW, ZR and ZRCW are circled into two zones, respectively. (Software of Adobe Illustrator CS 6, OriginPro 9.0, ArcGIS 10.2, and Microsoft Excel were used in drawing the figure. The outline of Shandong Province was drawn by using ArcGIS (version 10.2) and referring to the map from http://map.ps123.net/china/5369.html).

**Figure 2:**
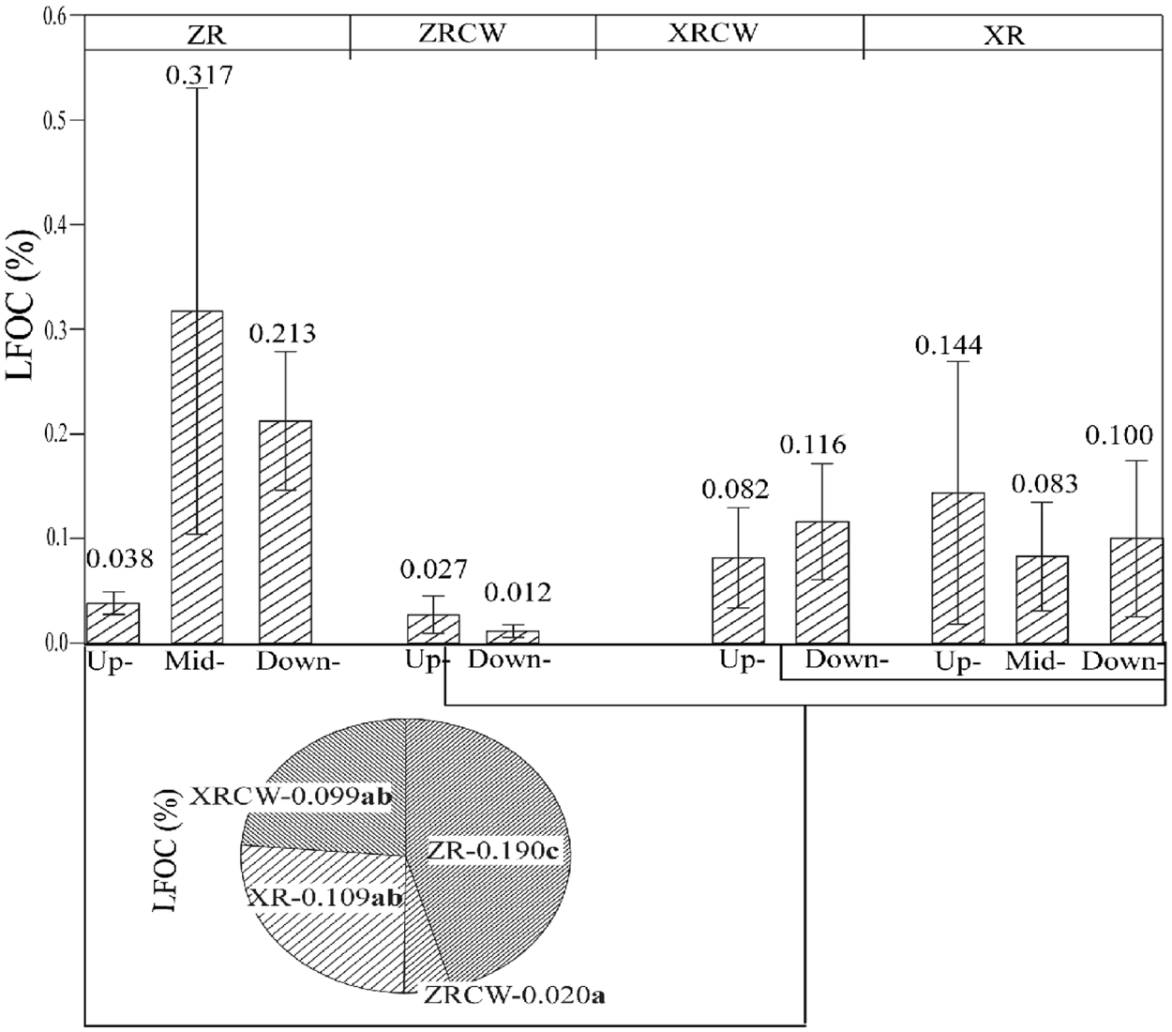
Cluster analysis and distributed difference of LFOC among the four wetlands. Up/Mid/Down-ZR: upstream/midstream/downstream of Zhaoniu River. Up/Down-ZRCW: upstream/downstream of Zhaoniu River Constructed Wetland. Up/Mid/Down-XR: upstream/midstream/downstream of Xinxue River. Up/Down-XRCW: upstream/downstream of Xinxue River Constructed Wetland. Data with the same lowercase letter (a or b) are not significant at α = 0.05 (Duncan test).

**Figure 3:**
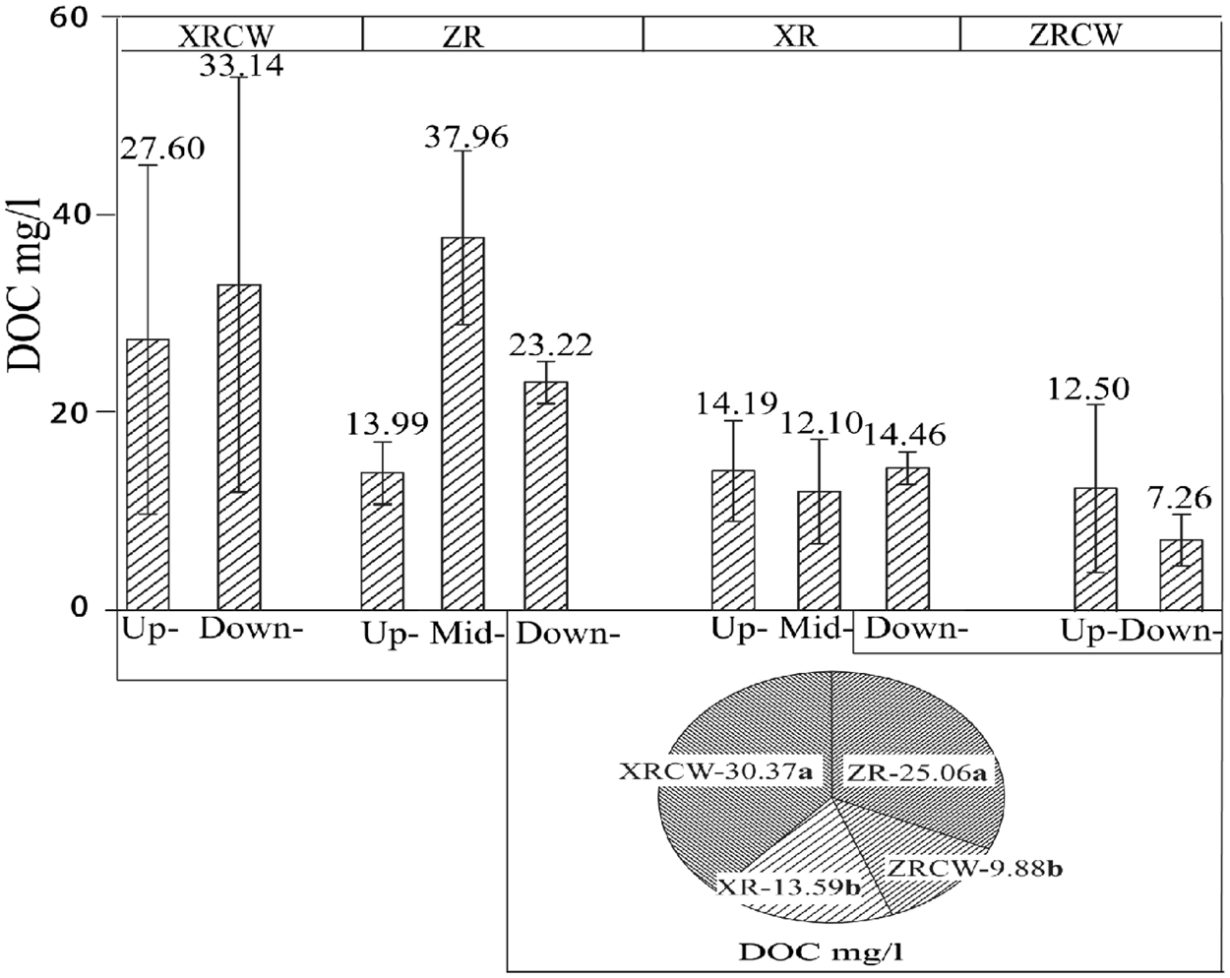
Cluster analysis and distributed difference of DOC among the four wetlands. Up/Mid/Down-ZR: upstream/midstream/downstream of Zhaoniu River. Up/Down-ZRCW: upstream/downstream of Zhaoniu River Constructed Wetland. Up/Mid/Down-XR: upstream/midstream/downstream of Xinxue River. Up/Down-XRCW: upstream/downstream of Xinxue River Constructed Wetland. Data with the same lowercase letter (a or b) are not significant at α = 0.05 (Duncan test).

Neither TOC nor HFOC exhibited any significant differences among two study zones (Fig. 1), two wetland types (constructed wetlands and river wetlands) or the four wetlands in Shandong Province of China (Fig. 1; Table 1). However, HFOC differed significantly among sampling stations in ZR and ZRCW and attained the highest value (3.072%) in downstream of ZR. Further, distribution of HFOC in XR and XRCW did not exhibit any significant differences (Fig. 1). River wetlands had significantly higher LFOC than those of the constructed wetlands (P = 0.012; Table 1 and Fig. 2), which may be mainly attributed to the significantly low LFOC in ZRCW (0.020% ± 0.01) and high LFOC in ZR (0.189% ± 0.17). Cluster analysis of LFOC showed that XR and XRCW had similar LFOC clustering. However, ZR and ZRCW had significantly different LFOC, which attributed to the high LFOC in Mid-ZR and Down-ZR (Fig. 2). The same clusters for DOC between XR and ZRCW and between ZR and XRCW showed that DOC distribution differed among different wetlands but not among wetland types (Fig. 3). Further, ZR and XRCW contributed most to DOC deposition. Both Mid- and Down-ZR had the same trend of LFOC and DOC, which was significantly higher than in Up-ZR and ZRCW (P = 0.000; Figs 2 and 3).

**Table 1:** Carbon contents and differences among the four wetlands (mean ± SD).

Organic carbon	Studied zones		Wetland types		Significance	
	ZR&ZRCW	XR&XRCW	River wetland	Constructed wetland	Zones*types	Four wetlands
**TOC (%)**	2.38 ± 0.64	2.56 ± 0.95	2.55 ± 0.79	2.35 ± 0.84	NA	NA
**HFOC (%)**	2.26 ± 0.55	2.45 ± 0.92	2.40 ± 0.73	2.29 ± 0.82	NA	NA
**LFOC (%)**	0.122 ± 0.15	0.105 ± 0.07	0.149 ± 0.14**a**	0.056 ± 0.05**b**	**	*
**DOC (mg/l)**	18.99 ± 12.2	20.30 ± 14.3	19.32 ± 10.3	20.12 ± 17.0	**	**

NA: no significant difference, *: P < 0.05, **: P < 0.01. Data with a and b show significant difference at α = 0.05 (Duncan test).

### Distribution differences of carbon fractions between two sampling campaigns

**Figure 4:**
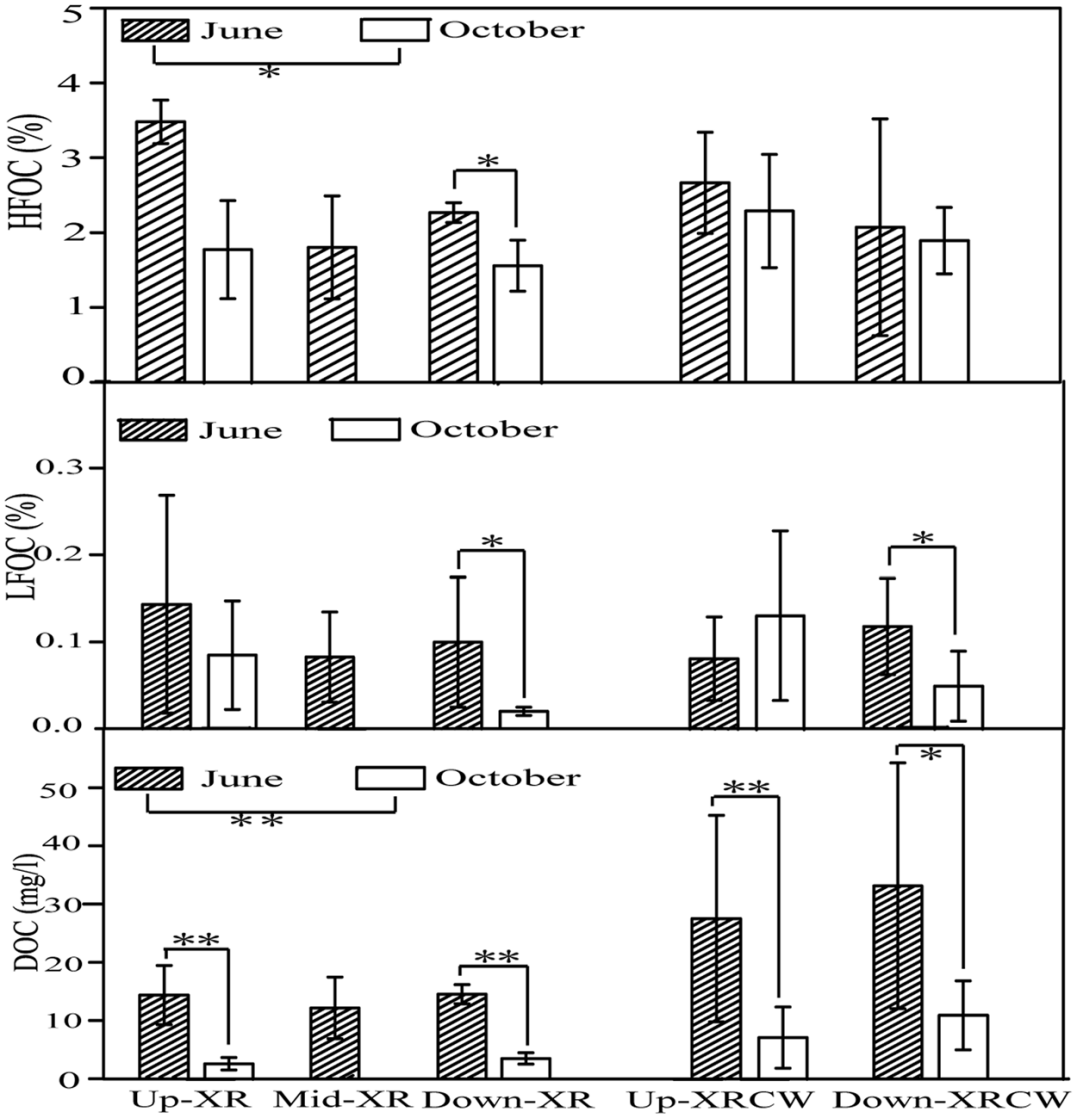
Seasonal differences of the carbon fractions (HFOC, LFOC, and LFOC) in Xinxue River (XR) and Xinxue River Constructed Wetland (XRCW). *P < 0.05, **P < 0.01 (Duncan test).

In general, carbon fractions in summer (June, 2014) had higher concentrations than those in autumn (October, 2015). Difference of HFOC was the least among the carbon fractions, whereas DOC was the largest (Fig. 4). LFOC in summer was higher or significantly higher than that in autumn except for Up-XRCW. One-way ANOVA analysis showed that XR and XRCW had significantly higher HFOC (P = 0.039) and DOC (P = 0.000) in the summer than in the autumn by analyzing all the sampling stations though the difference between certain stations may not significant (for example, the HFOC of Up- and Down-XRCW in summer and autumn are similar). Total organic carbon (TOC; 2.56% in summer & 2.06% in autumn) in summer was significantly higher than in autumn (P = 0.039).

### Physical and microbial factors affecting carbon deposition

**Figure 5:**
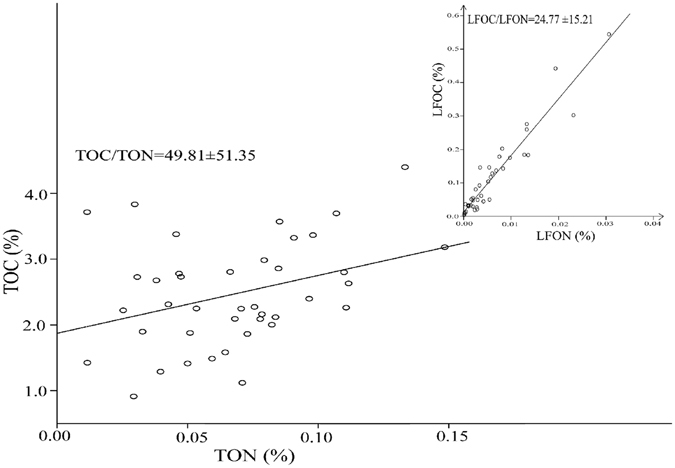
Linear regression analyses of total organic carbon (TOC) and total organic nitrogen (TON), and of light fraction organic carbon (LFOC) and light fraction organic nitrogen (LFON). R^2^_(TOC/TON_) = 0.122 (P = 0.027); R^2^_(LFOC/LFON)_ = 0.907 (P = 0.000).

Moisture content and bulk density were significantly correlated to HFOC, heavy fraction organic nitrogen (HFON; the concentration of organic nitrogen in the separated heavy fraction, which was described in the “Material and Method” section) and DOC, while they did not have a marked effect on LFOC and light fraction organic nitrogen (LFON; Pearson correlation analysis from the Supplementary file). A prominent linear relationship existed between LFOC and LFON (R^2^ = 0.907, P = 0.000), with the mean value (24.77) of LFOC to LFON ratio, which was lower than TOC to TON ratio (49.81) (Fig. 5). And Principle Component Analysis showed that moisture is very close to LFOC, LFON, and DOC (Supplementary file). C and N fractions were strongly associated with each other, except for HFOC and HFON (Supplementary file). In general, carbon input also had a complex relationship with microbes. In the present study, *Acidobacteria-6* was positively associated with LFOC (P < 0.01) in ZR and ZRCW, and *Bacteroidetes* was negatively correlated with HFON (P < 0.05; Supplementary file). *Thiobacillus*, *Burkholderiales* and *Rhodocyclales* were all positively associated with carbon fractions and LFON in XR and XRCW. Specific Pearson correlation analysis between microbial communities and carbon and nitrogen fractions were showed in the Supplementary file.

### Distribution patterns of carbon deposition at national scale

**Figure 6:**
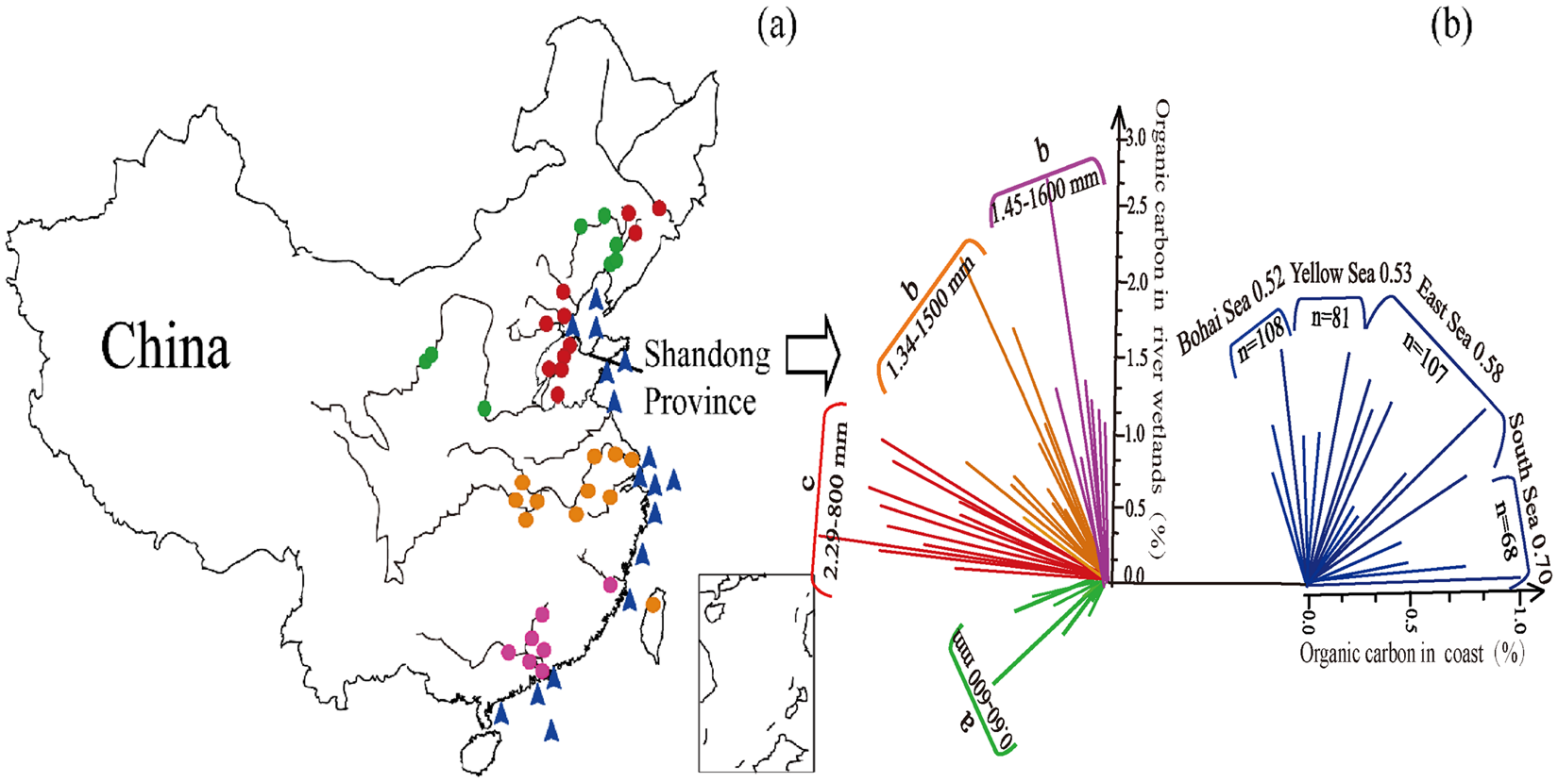
National distribution of sampling stations and organic carbon contents. Map shows the: (**a**) national sampling stations including inland and coastal areas, and (**b**) organic carbon contents of inland and coastal areas with different precipitation. Colors of (**a**) and (**b**) are similar in both a and b maps^4, 18–51^. Areas followed by the same lowercase letters (**a**, **b** and **c**) are not significant at α = 0.05 (Duncan test). (Software of Adobe Illustrator CS 6, OriginPro 9.0, ArcGIS 10.2, and Microsoft Excel were used in drawing the figure. The outline of China was drawn by using ArcGIS (version 10.2) and referring to the map from http://map.ps123.net/china/5369.html).

River sediments were studied for four precipitation regions (600 mm, 800 mm, 1500 mm, and 1600 mm) and three climatic zones (cold temperature zone, north subtropical zone, and edge subtropical zone). The OC concentration differed significantly among the precipitation regions (P = 0.000; Fig. 6 and Table 2), and attained the maximum value (2.29%) corresponding with the annual precipitation of 800 mm, and it was significantly higher than OC concentrations (0.60%) with the precipitation of 600 mm. However, river OC with the mean value of 1.58% across China, did not differ among the three climate zones in (P = 0.272). Google Scholar, covering most studies on OC across China, showed that most studies of the sediment OC are confined to the eastern China. OC in river sediments (1.58%) was significantly higher than that in the marine sediments (P = 0.000; 0.59%), and follows an increasing trend with the decrease in the latitude (Table 2).

**Table 2:** The concentrations of organic carbon in the four precipitation regions, three climate zones, and four coastal seas.

Precipitation	Number of samples	Organic carbon (%)
**600 mm**	153	0.60% **a** ± 0.43
**800 mm**	167	2.29% **c** ± 0.50
**1500 mm**	157	1.34% **b** ± 0.61
**1600 mm**	118	1.45% **b** ± 0.64
**Climate zone**	**Number of samples**	**Organic carbon (%)**
**Cold temperature zone**	219	1.82% ± 0.87
**North subtropical zone**	151	1.38% ± 0.63
**Edge subtropical zone**	124	1.39% ± 0.62
**Coastal sea**	**Number of samples**	**Organic carbon (%)**
**Bohai Sea**	108	0.52% ± 0.17
**Yellow Sea**	81	0.53% ± 0.15
**East Sea**	107	0.58% ± 0.22
**South Sea**	68	0.70% ± 0.23

Data with a, b, and c show significant difference at α = 0.05 (Duncan test).

## Discussion

Carbon: nitrogen ratio is an important factor which can be used to distinguish carbon sources from aquatic plants or terrestrial plants^52^. Meyers (1994) reported that carbon deposition in soils or sediments is primarily derived from terrestrial plants if the C: N ratio is >20^53^. Therefore, the high C: N ratio observed in the present study (TOC/TON = 49.81; LFOC/LFON = 24.77) suggests that OC input is primarily from the terrestrial plants. Goldman *et al*. (1987) reported that 53:6 (or 8.83:1) is the optimum C: N ratio for microbial growth, and any deviation from this ratio would strongly limit the microbial growth^54^. Tan *et al*.^6^ and Xiang *et al*.^55^ also reported that microbial biomass carbon to nitrogen ratio is about 9.5:1 and 23:3 ( = 7.7:1), respectively. Thus, the high LFOC: LFON ratio observed in the present study may indicate that the C is primarily derived from the terrestrial plants rather than from the microbial residues. In addition, the significantly higher TOC: TON ratio in ZRCW (70.6:1) and up-XR (141.8:1) than that in other stations in the present study indicate that low N concentration limits microbial growth and activities. Furthermore, it could also limit C accumulation but increase C mineralization. These results are similar to those of Moore *et al*.^56^ who also observed that unbalanced C: N ratio can lead to N fixation but C mineralization until the dynamic balance is achieved. Therefore, under conditions of N insufficiency, available N sequestration is an important factor affecting the C deposition.

Carbon storage from the above ground plants into sediments immediately impacts the microbial activities^57^. Elshahed *et al*. (2007) reported that *Acidobacteria-6* impacts both carbon deposition and ammonia oxidation in sediments or soils^58^. Further, such a relationship is also observed between LFOC, HFON, DOC and *Acidobacteria-6* in ZR and ZRCW. However, the negative correlation between HFON and *Bacteroidales* may suggest that activities of *Bacteroidales* limit the deposition of HFON in the present study^13^.

The stable HFOC^5^, which accounts for 95.4% of total OC, leads to the non-significant differences of OC distribution among the studied four wetlands. Whereas the interaction effect between study zones and wetland types causes the prominent differences of LFOC and DOC distribution (Table 1). Therefore, LFOC and DOC are relatively sensitive to environmental changes, which is in accord with the reports of Tan *et al*.^6^. The lower DOC and LFOC in down-ZR and ZRCW than these in up-ZR and mid-ZR may be associated with the low concentration of dissolved oxide (DO, ranging from 0.0 to 0.7 mg l^−1^) and subsequently the microbial communities. Fasching *et al*.^59^ reported that DOC can influence the microbial activities, whereas Jiao *et al*. (2010) showed that dissolved organic matter (DOM) can be mineralized by microbes^60^. Therefore, DOC can be regulated and limited by microbes to some extent. LFOC is primarily related to land use, plant coverage^61, 62^, microbial activities^63^ and C mineralization^64^. XRCW, with significantly higher plant coverage and species and microbial diversity than XR, whereas XRCW has similar level of LFOC with XR. Thus, the data presented herein show that plant coverage and microbial activities may be not the determining factors for LFOC. However, for LFON, which had very low content in the sediments, is significantly correlated with LFOC (R^2^ = 0.907; Fig. 5). So LFOC deposition from terrestrial plants into wetland sediments is mainly limited by LFON in this study. This finding can also explain the result of Lal (2005) that increased plant litter may not necessarily raise the carbon storage^65^.

The four seasons of a year, with different climates, air temperatures, water level fluctuations and precipitations, have strong impacts on C deposition and emission^66^. The data reported herein show that higher carbon fractions (HFOC, LFOC, and DOC) in June than those in October. This result further suggests that summer has higher C storage than autumn. Whereas Sabrekov *et al*. (2014) proved that emission of greenhouse gases (GHGs) is mostly during the summer^67^. In addition, Xu *et al*. (2015) observed that CH_4_ content in summer of XRCW is 15.5 times higher than that in autumn^14^. Therefore, summer is an important season to assess whether wetland is a carbon source or sink. Emission of GHGs is also strongly affected by plant species and the relative surface covered^66, 68^. Xu *et al*. (2014) also showed that GHGs emission in mud flat, which has no covered plants, exhibited no significant difference among seasons^66^. Thus, notably higher plant coverage and species in XRCW than in XR may contribute to the higher potential for GHGs emission in summer in XRCW than XR, and was also easily to be carbon source.

Concentration of OC in worldwide natural wetlands (22.92 mol kg^−1^) is significantly higher than that in river wetlands of China (1.58% ± 0.011)^9^, so is the OC density (8.01 kg C/m^2^ in China to 10.60 kg C/m^2^ in the world)^69^. Lal (2004) reported that soil physical structure can affect the carbon sequestration significantly^70^. Therefore, the prominent relationships among HFOC, DOC and bulk density herein may indicate that soil structure significantly affects the deposition of HFOC and DOC but not the LFOC in the present study. Above the ground, carbon sequestration may also be influenced by plants and carbon dioxide (CO_2_) in atmosphere. Mitsch *et al*. (2014) reported that plant richness in wetland can notably increase carbon sequestration compared to increasing methane (CH_4_) emission^15^; Van Groenigen *et al*. (2011, 2014) also reported that with elevated CO_2_ concentration in atmosphere, increased microbial decomposition rate and CH_4_ emission in natural wetland limits carbon sequestration process^71, 72^. Thus, the significantly lower carbon sequestration in natural wetland of China than the world’s mean level suggests that Chinese natural wetlands still have great potential for carbon sequestration. Effective measures should be carried out to mitigate the increasing CO_2_ concentration in China^73^. Google Scholar showed that most field studies on organic carbon of ecosystem in China focused on the eastern China, where concentrated with primarily population, industries, precipitation, cultivated land and river wetland^17^. Semi-arid grassland soils (such as northern China) can also accumulate stable organic carbon without much land use^73, 74^. In China, effective management and proper protection on semi-arid grassland may improve higher carbon sequestration than that on the eastern land, which endured substantially disturbance. Consequently, these management and protection to terrestrial ecosystems are essential to carbon storage in national level.

Three climatic zones and four precipitation regions, which were divided by Shi *et al*. (2013) and Wang *et al*. (2014)^16, 74^, were involved to analyze OC storage in this study. The significant different OC distribution across among precipitation regions and the non-significant OC distribution across the sampled four wetlands suggested that precipitation is one important factor affecting carbon storage in large scale. The higher carbon storage in precipitation about 800 mm than those about 600 mm, 1500 mm or 1600 mm suggested that proper precipitation prone to carbon storage. Areas with precipitation of 1500 and 1600 mm had relative high temperature. And Bauer *et al*.^75^ showed that dry sites are more inclined to be carbon sink than humid sites in tropical. A significant logistic relationship showed that OC increased with an increase in precipitation and moisture to some extent^76^. Therefore, the too much precipitation may go against the carbon storage. Carbon deposition trend among the climatic zones of China is also similar with the report of Bauer *et al*.^75^, who showed higher carbon sequestration in temperate wetland than in tropical and boreal wetland by balancing CO_2_ sink and CH_4_ emission. Fine fraction of soils prone to carbon accumulation^73^ and high temperature and water content would increase microbial decomposition rate to plant residues^72^. Therefore, OC differences among different climatic zones and precipitations may be also induced by proportions of particle sizes in sediments and microbial richness. The notably lower OC in marine sediments than inland river wetland may suggest that carbon accumulation in ocean is less than in river wetland. The OC distribution trend, increasing with the reduced latitude, differs from carbon distribution in river wetland. One source of the OC in coastal sediments is the water flow from terrestrial rivers^77^. The particle fluxes from terrestrial river into coast are related to the terrain, runoff amount, and other environmental conditions^78^. And Ni *et al*. (2008) also reported that the maximum fluxes of suspended particle are in coincidence with the largest precipitation^79^. However, specific contributing factors should be studied in further research.

In conclusion, the hypothesis we established is proved in this study, and the results presented support the following conclusions: (1) Sampling season can affect the storage of carbon fractions in temporal scale significantly; (2) Imbalanced C: N ratio could hinder the carbon sequestration in wetland in regional scale; (3) Proper precipitation is beneficial to carbon deposition in large scale, and carbon storage in river wetlands is prominently higher than in the coastal China; (4) However, the effects of wetland types and climatic zones on OC storage is not prominent in the present study. Therefore, comprehensive work should to be done to further confirm the influence of wetland types and climate on OC deposition, and global studies on carbon storage are also needed in the next step.

## Materials and Methods

### Field sampling and data collection

Sediments were sampled from four wetlands (Zhaoniu River (ZR) and Zhaoniu River Constructed Wetland (ZRCW), Xinxue River (XR) and Xinxue River Constructed Wetland (XRCW)) in Shandong Province in the northern China. The factors affecting wetland C storage in regional scale were analyzed. ZR and XR are two tributaries of Tuhai River and Nansi Lake, respectively (Fig. 1). Nansi Lake is one of the largest lakes in the South-to-North Water Transfer Project and Tuhai River is one important river of the Haihe River Basin. The ZRCW and XRCW were constructed in 2012 and 2007, respectively^51^, (Fig. 1) on the Tuhai River and Nansi Lakes to control pollution by the domestic sewage and industrial wastewater of cities.

Surface sediments were sampled in June 2015 from upstream, midstream, and downstream of ZR and XR; and upstream and downstream of ZRCW and XRCW. A total of 40 sediment samples (34°32′-34°48′N; 117°08′-117°15′E) were collected from the four wetlands (Fig. 1) to analyze the distribution of the three C fractions. The OC deposition was assessed in October 2015 in XR and XRCW to compare seasonal differences among C fractions^4^.

China is a fast developing country^80^, and papers on OC published before 2006 mostly concentrated in terrestrial systems which suffered serious environmental damage^81, 82^. In addition, part of the methods to determine the OC ten years ago was not as accurate as the method we use in recent years^83^ (Potassium dichromate external heating method and the element analyzer method). Thus, data published before 2006 are excluded from this research. Previous studies also reported that the concentration of OC in surface soils and sediments was higher than in subsurface or deep soils^11^. And OC in deep soils was relatively stable and not easy to be affected by environmental factors^84^. So sediments deeper than 30 cm were also excluded from the present study. To make sure the data we retrieved is reliable, the published papers those have high cited times are referred firstly. The specific process of data retrieve is listed below.

“The OC in river sediments of China” was searched with Google Scholar (http://scholar.glgoo.org/), and the first 100 publications (listed by correlations high to low;) and the data were screened for the following requirements: 1) the articles published after 2006; 2) the data of sediments sampled before 2000 were eliminated; 3) sediment samples of deeper than 30 cm were excluded from the collected data; 4) the research stations not relevant to river wetlands or coastal wetlands were eliminated; 5) the data reused in two or more publications were retrieved only once; 6) the OC contents which could specifically be transformed into percentile system were retrieved; and 7) the OC determined by elemental analyzer was used to avoid experimental error. Finally, 595 data from river sediment samples and 364 data from coastal sediment samples published in 38 articles were retrieved for this study. In total, sediment data of over 40 rivers and tributaries and four coastal seas (Bohai Sea, Yellow Sea, East Sea, and South Sea) were used in the present study. The sampling stations and C distributions are described in Fig. 2. In addition, data on precipitations, climate zones and land-sea differences were also obtained to assess the distribution trend of OC across China^16^.

### Laboratory analyses

Prior to further analysis, moisture content and bulk density were calculated by comparing the volume of sediment samples under room temperature and 105 °C^85^. Sediment samples were air dried, ground and sieved through 2 mm at room temperature (~20 °C) for extraction of DOC^86, 87^. The concentration of DOC was measured by a total-C analyzer (TOC-L CPN, Shimadzu, Japan) using a non-purgeable OC analysis procedure. The pH was measured in 1:2.5 sediment: water suspension. The 1.70 g mL^−1^ of sodium iodide solution was used to separate heavy fraction organic matter (HFOM) and light fraction organic matter (LFOM) from sediment samples^84^. LFOM and HFOM were weighed by an electronic balance (0.0000 g), and C and N contents (LFOC, LFON, HFOC, and HFON) were determined by an elemental analyzer (Vario EL III, Elementar Analysensysteme, Germany). Total carbon to nitrogen ratio (TC/TN), light fraction carbon to nitrogen ratio (LFOC/LFON), and heavy fraction carbon to nitrogen ratio (HFOC/HFON) were calculated for further analysis.

The analyses of DNA extraction and Illumina MiSeq sequencing of the amplified DNA were conducted at Shanghai Paisennuo Biological Technology Co. Ltd (Shanghai, China). Microbial communities and populations were cited and analyzed to explain the distribution of C fractions^88^.

### Statistical analyses

Statistical analyses of the data were performed by using the SPSS 21.0. Mean value analysis and one-way analysis of variance (ANOVA) were computed to compare the differences of OC in inland rivers and sea areas of China. In addition, mean value analysis, one-way and two-way ANOVA, cluster analysis and correlation analysis were performed for the data on the carbon fractions. Cluster analysis to LFOC and DOC: mean values of LFOC and DOC in the four wetlands (ZR, ZRCW, XR, and XRCW) are as four variables for cluster analysis to LFOC or DOC. Moreover, linear-regression analysis in SPSS and Principal component analysis (PCA) in Canoco 4.5 were performed between C fractions and other characteristics of the sediments (pH, moisture, bulk density, and nitrogen fractions). Correlation analysis was also done between C, N fractions and main microbial taxonomies. Origin 9, ArcGIS 10.2 and Adobe Illustrator (version 16.0.0) were used to draw figures.

### Ethics Statement

The sample collection of our study was conducted with the official permission of the Environmental Protection Bureau of Weishan Country and the Xinxue River Constructed Wetland Management Committee.

## Electronic supplementary material

Supplementary file
