# Supplementary material for: Factors affecting distribution patterns of organic carbon in sediments at regional and national scales in China
Source: Sci Rep. 2017 Jul 14;7:5497. doi: 10.1038/s41598-017-06035-z (PMC5511284; doi:10.1038/s41598-017-06035-z)
Supplement: Supplementary file 1 — Supplementary file [file 41598_2017_6035_MOESM1_ESM.pdf]

Factors affecting distribution patterns of organic carbon in sediments at regional and national scale in China

Qingqing Cao[1, 2], Hui Wang[3], Yiran Zhang[4], Rattan Lal[2], Renqing Wang[1, 3, 5], Xiuli Ge[6], Jian Liu[1*]

1. Institute of Environmental Research, Shandong University, Jinan 250100, China

2. Carbon Management and Sequestration Center, The Ohio State University, Columbus, 43210, USA

3. School of Life Sciences, Shandong University, Jinan 250100, China

4. Shenyang Academy of Environmental Sciences, Shenyang 110167, China

5. Shandong Provincial Engineering and Technology Research Center for Vegetation Ecology, Shandong University, Jinan 250100, China

6. School of Environmental Science and Engineering, Qilu University of Technology, Jinan, 250353, China

*Corresponding author: Jian Liu,

Institute of Environmental Research,

Shandong University, Jinan 250100, China;

Tel: +86 531 88364425;

Fax: +86 531 88361990;

E-mail: ecology@sdu.edu.cn

Supplementary files

Supplementary file 1

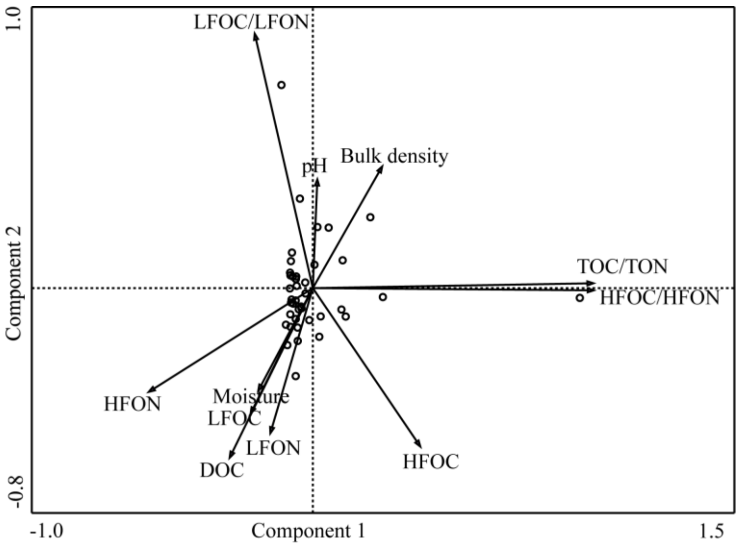

Figure S1. Principle Component Analysis (PCA) to carbon fractions, physical and microbial factors.

Supplementary file 2

P	HFOC	LFOC	HFON	LFON	DOC	pH	Moisture
**HFOC**	1						
**LFOC**	0.328*	1					
**HFON**	0.201	0.562**	1				
**LFON**	0.413**	0.952**	0.511**	1			
**DOC**	0.424**	0.615**	0.723**	0.538**	1		
**pH**	-0.406**	-0.08	0.065	-0.094	-0.276	1	
**Moisture**	0.447**	0.096	0.424**	0.14	0.635**	-0.429**	1
**Bulk density**	-0.409**	-0.081	-0.393*	-0.174	-0.505**	0.454**	-0.888**

Table S1. The correlation analysis among carbon fractions, nitrogen fractions and soil physical parameters. *: P<0.05, **: P<0.01.

Supplementary file 3

P	HFOC	LFOC	HFON	LFON	DOC	pH	Moisture	Bulk density
Acidobacteria		0.506**	0.615**	0.379*	0.396*			
Bacteroidetes			-0.624**					0.325*
Proteobacteria			0.362*					
Euryarchaeota							0.523**	-0.497**
Thiobacillus			0.334*			0.404**		
Desulfobacterales					0.384*	-0.373*	0.514**	-0.481**
Syntrophobacterales		0.380*	0.596**		0.596**			
Bacteroidales			-0.385*			-0.471**		
Flavobacteriales			-0.653**		-0.328*			0.343*
Acidobacteria6		0.501**	0.619**	.370*	0.395*			
Pirellulales		0.403**						
Methanomicrobia							0.502**	-0.480**

Table S2. The Correlation analysis between microbial orders and carbon-nitrogen fractions in the four wetlands. *: P<0.05, **: P<0.01.
